# Diaqua­bis­(2-bromo­benzoato-κ*O*)bis­(*N*,*N*-diethyl­nicotinamide-κ*N* ^1^)cobalt(II)

**DOI:** 10.1107/S1600536810032630

**Published:** 2010-08-18

**Authors:** Tuncer Hökelek, Güner Saka, Barış Tercan, Füreya Elif Öztürkkan, Hacali Necefoğlu

**Affiliations:** a Department of Physics, Hacettepe University, 06800 Beytepe, Ankara, Turkey; b Department of Chemistry, Hitit University, 19030 Ulukavak, Çorum, Turkey; c Department of Physics, Karabük University, 78050 Karabük, Turkey; d Department of Chemistry, Kafkas University, 36100 Kars, Turkey

## Abstract

In the mononuclear title compound, [Co(C_7_H_4_BrO_2_)_2_(C_10_H_14_N_2_O)_2_(H_2_O)_2_], the Co^II^ ion is located on a crystallographic inversion center. The asymmetric unit is completed by one 2-bromo­benzoate anion, one diethyl­nicotinamide (DENA) ligand and one coordinated water mol­ecule; all ligands are monodentate. The four O atoms in the equatorial plane around Co^II^ form a slightly distorted square-planar arrangement, while the slightly distorted octa­hedral coordination is completed by the two pyridine N atoms of the DENA ligands in axial positions. The dihedral angle between the carboxyl­ate group and the attached benzene ring is 84.7 (1)°; the pyridine and benzene rings are oriented at a dihedral angle of 43.64 (6)°. In the crystal structure, inter­molecular O—H⋯O and C—H⋯O hydrogen bonds link the mol­ecules into a three-dimensional network.

## Related literature

For niacin, see: Krishnamachari (1974 ▶). For *N*,*N*-diethyl­nicotinamide, see: Bigoli *et al.* (1972 ▶). For related structures, see: Hökelek, Dal, Tercan, Aybirdi *et al.* (2009 ▶); Hökelek *et al.* (2009*a* ▶,*b* ▶,*c* ▶,*d* ▶,*e* ▶); Necefoğlu *et al.* (2010 ▶).
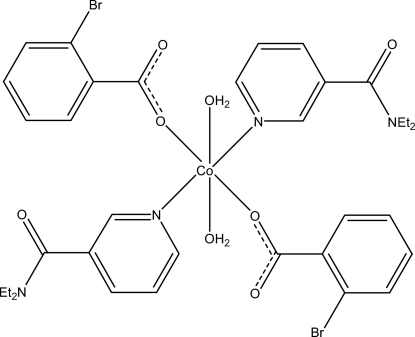


## Experimental

### 

#### Crystal data

[Co(C_7_H_4_BrO_2_)_2_(C_10_H_14_N_2_O)_2_(H_2_O)_2_]
*M* *_r_* = 851.43
Monoclinic, 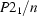

*a* = 13.0106 (2) Å
*b* = 10.3513 (2) Å
*c* = 14.9580 (3) Å
β = 114.311 (1)°
*V* = 1835.86 (6) Å^3^
*Z* = 2
Mo *K*α radiation
μ = 2.70 mm^−1^
*T* = 100 K
0.31 × 0.28 × 0.23 mm

#### Data collection

Bruker Kappa APEXII CCD area-detector diffractometer
Absorption correction: multi-scan (*SADABS*; Bruker, 2005 ▶) *T*_min_ = 0.489, *T*_max_ = 0.576
16787 measured reflections
4528 independent reflections
3700 reflections with *I* > 2σ(*I*)
*R*_int_ = 0.029

#### Refinement

*R*[*F* ^2^ > 2σ(*F* ^2^)] = 0.028
*wR*(*F* ^2^) = 0.064
*S* = 1.04
4528 reflections
233 parameters
H atoms treated by a mixture of independent and constrained refinement
Δρ_max_ = 0.60 e Å^−3^
Δρ_min_ = −0.32 e Å^−3^

### 

Data collection: *APEX2* (Bruker, 2007 ▶); cell refinement: *SAINT* (Bruker, 2007 ▶); data reduction: *SAINT*; program(s) used to solve structure: *SHELXS97* (Sheldrick, 2008 ▶); program(s) used to refine structure: *SHELXL97* (Sheldrick, 2008 ▶); molecular graphics: *ORTEP-3 for Windows* (Farrugia, 1997 ▶) and *PLATON* (Spek, 2009 ▶); software used to prepare material for publication: *WinGX* (Farrugia, 1999 ▶) and *PLATON*.

## Supplementary Material

Crystal structure: contains datablocks I, global. DOI: 10.1107/S1600536810032630/im2218sup1.cif

Structure factors: contains datablocks I. DOI: 10.1107/S1600536810032630/im2218Isup2.hkl

Additional supplementary materials:  crystallographic information; 3D view; checkCIF report

## Figures and Tables

**Table 1:** Hydrogen-bond geometry (Å, °)

*D*—H⋯*A*	*D*—H	H⋯*A*	*D*⋯*A*	*D*—H⋯*A*
O4—H41⋯O3^i^	0.82 (3)	1.94 (3)	2.757 (2)	174 (3)
O4—H42⋯O1^ii^	0.78 (3)	1.90 (3)	2.644 (2)	159 (2)
C4—H4⋯O1^iii^	0.93	2.56	3.184 (3)	125
C10—H10⋯O2^iv^	0.93	2.44	3.368 (2)	174
C12—H12⋯O3^v^	0.93	2.33	3.259 (2)	179
C16—H16*A*⋯O3^vi^	0.97	2.57	3.506 (2)	163
C16—H16*B*⋯O1^i^	0.97	2.52	3.460 (3)	164

Symmetry codes: (i) 
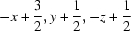
; (ii) 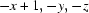; (iii) 
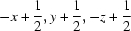
; (iv) 
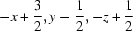
; (v) 
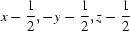
; (vi) 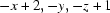.

**Fig. 1.**
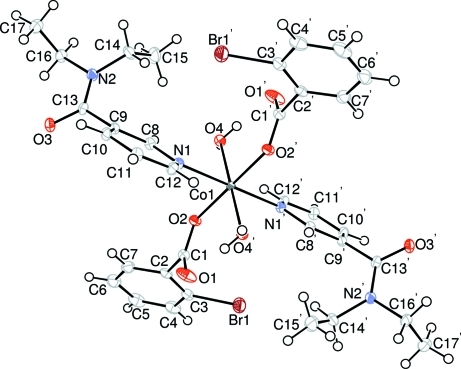
The molecular structure of the title compound, showing the atom-numbering scheme. Displacement ellipsoids are drawn at the 50% probability level. [Symmetry code: (') 1-x, -y, -z.]

**Fig. 2.**
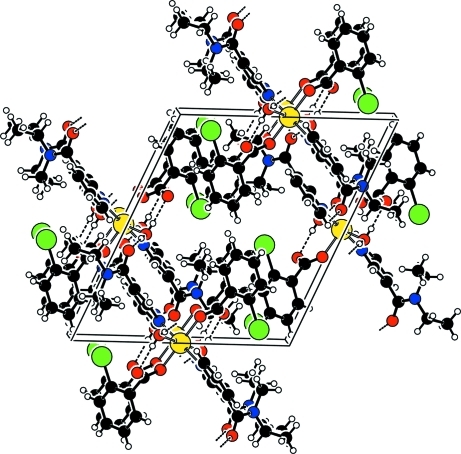
Partial packing diagram, viewed down the baxis, with the aaxis horizontal and the c axis vertical. Hydrogen bonds are shown as dashed lines.

## supplementary crystallographic information

### Comment

As a part of our ongoing investigation on transition metal complexes of nicotinamide (NA), one form of niacin (Krishnamachari, 1974), and/or the nicotinic acid derivative *N*,*N*-diethylnicotinamide (DENA), an important respiratory stimulant (Bigoli *et al.*, 1972), the title compound was synthesized and its crystal structure is reported herein.

The title compound, (I), is a mononuclear complex, in which the Co^II^ ion is located on a crystallographic inversion center. The asymmetric unit contains one 2-bromobenzoate (BB) anion, one diethylnicotinamide (DENA) ligand and one coordinated water molecule, all ligands are monodentate (Fig. 1). The crystal structures of some DENA complexes of Co^II^, Ni^II^, Mn^II^ and Zn^II^ ions, [Co(C_8_H_7_O_2_)_2_(C_10_H_14_N_2_O)_2_(H_2_O)_2_], (II) (Necefoğlu *et al.*, 2010), [Co(C_9_H_10_NO_2_)_2_(C_10_H_14_N_2_O)_2_(H_2_O)_2_], (III) (Hökelek, Dal, Tercan, Aybirdi *et al.*, 2009), [Ni(C_7_H_4_ClO_2_)_2_(C_10_H_14_N_2_O)_2_(H_2_O)_2_], (IV) (Hökelek *et al.*, 2009*a*), [Ni(C_7_H_4_BrO_2_)_2_(C_10_H_14_N_2_O)_2_(H_2_O)_2_], (V) (Hökelek *et al.*, 2009*e*), [Mn(C_7_H_4_BrO_2_)_2_(C_10_H_14_N_2_O)_2_(H_2_O)_2_], (VI) (Hökelek *et al.*, 2009*b*), [Mn(C_7_H_4_ClO_2_)_2_(C_10_H_14_N_2_O)_2_(H_2_O)_2_], (VII) (Hökelek *et al.*, 2009*c*) and [Zn(C_7_H_4_BrO_2_)_2_(C_10_H_14_N_2_O)_2_(H_2_O)_2_], (VIII) (Hökelek *et al.*, 2009*d*) have been reported before.

The four O atoms (O2, O4 and symmetry-related atoms O2', O4') in the equatorial plane around the Co^II^ ion form a slightly distorted square-planar arrangement. The slightly distorted octahedral coordination is completed by the two pyridyl N atoms of DENA ligands (N1, N1') in axial positions (Fig. 1). The near equality of C1—O1 [1.242 (2) Å] and C1—O2 [1.264 (2) Å] in the carboxylate group indicates a delocalized bonding arrangement rather than localized single and double bonds. The average Co—O bond length is 2.092 (1) Å (Table 1), and the Co^II^ ion is displaced out of the least-squares plane of the carboxylate group (O1/C1/O2) by -0.3436 (1) Å. The dihedral angle between the planar carboxylate moiety and the benzene ring A (C2—C7) is 84.7 (1)°, while that between rings A and B (N1/C8—C12) is 43.64 (6)°.

In the crystal structure, intermolecular O—H···O and C—H···O hydrogen bonds (Table 2) link the molecules into a three-dimensional network (Fig. 2).

### Experimental

The title compound was prepared by the reaction of CoSO_4_ × 7 H_2_O (1.40 g, 5 mmol) in H_2_O (40 ml) and diethylnicotinamide (1.78 g, 10 mmol) in H_2_O (20 ml) with sodium 2-bromobenzoate (2.23 g, 10 mmol) in H_2_O (50 ml). The mixture was filtered and set aside to crystallize at ambient temperature for two weeks, giving pink single crystals (yield; 3.065 g, 72%).

### Refinement

Atoms H41 and H42 (for H_2_O) were located from a difference Fourier map and refined isotropically. The remaining H atoms were positioned geometrically with C—H = 0.93, 0.97 and 0.96 Å for aromatic, methylene and methyl H atoms and constrained to ride on their parent atoms, with *U*_iso_(H) = *xU*_eq_(C), where *x* = 1.5 for methyl H and *x* = 1.2 for all other H atoms.

### Crystal data

**Table:**

[Co(C_7_H_4_BrO_2_)_2_(C_10_H_14_N_2_O)_2_(H_2_O)_2_]	*F*(000) = 866
*M**_r_* = 851.43	*D*_x_ = 1.540 Mg m^−^^3^
Monoclinic, *P*2_1_/*n*	Mo *K*α radiation, λ = 0.71073 Å
Hall symbol: -P 2yn	Cell parameters from 6465 reflections
*a* = 13.0106 (2) Å	θ = 2.5–28.3°
*b* = 10.3513 (2) Å	µ = 2.70 mm^−^^1^
*c* = 14.9580 (3) Å	*T* = 100 K
β = 114.311 (1)°	Block, pink
*V* = 1835.86 (6) Å^3^	0.31 × 0.28 × 0.23 mm
*Z* = 2	

### Data collection

**Table:**

Bruker Kappa APEXII CCD area-detector diffractometer	4528 independent reflections
Radiation source: fine-focus sealed tube	3700 reflections with *I* > 2σ(*I*)
graphite	*R*_int_ = 0.029
φ and ω scans	θ_max_ = 28.4°, θ_min_ = 1.8°
Absorption correction: multi-scan (*SADABS*; Bruker, 2005)	*h* = −17→17
*T*_min_ = 0.489, *T*_max_ = 0.576	*k* = −13→13
16787 measured reflections	*l* = −19→17

### Refinement

**Table:**

Refinement on *F*^2^	Primary atom site location: structure-invariant direct methods
Least-squares matrix: full	Secondary atom site location: difference Fourier map
*R*[*F*^2^ > 2σ(*F*^2^)] = 0.028	Hydrogen site location: inferred from neighbouring sites
*wR*(*F*^2^) = 0.064	H atoms treated by a mixture of independent and constrained refinement
*S* = 1.04	*w* = 1/[σ^2^(*F*_o_^2^) + (0.0277*P*)^2^ + 0.7345*P*] where *P* = (*F*_o_^2^ + 2*F*_c_^2^)/3
4528 reflections	(Δ/σ)_max_ < 0.001
233 parameters	Δρ_max_ = 0.60 e Å^−^^3^
0 restraints	Δρ_min_ = −0.32 e Å^−^^3^

### Special details

**Table:**

Geometry. All esds (except the esd in the dihedral angle between two l.s. planes) are estimated using the full covariance matrix. The cell esds are taken into account individually in the estimation of esds in distances, angles and torsion angles; correlations between esds in cell parameters are only used when they are defined by crystal symmetry. An approximate (isotropic) treatment of cell esds is used for estimating esds involving l.s. planes.
Refinement. Refinement of F^2^ against ALL reflections. The weighted R-factor wR and goodness of fit S are based on F^2^, conventional R-factors R are based on F, with F set to zero for negative F^2^. The threshold expression of F^2^ > 2sigma(F^2^) is used only for calculating R-factors(gt) etc. and is not relevant to the choice of reflections for refinement. R-factors based on F^2^ are statistically about twice as large as those based on F, and R- factors based on ALL data will be even larger.

### Fractional atomic coordinates and isotropic or equivalent isotropic displacement parameters (Å^2^)

**Table:**

	*x*	*y*	*z*	*U*_iso_*/*U*_eq_	
Br1	0.178480 (15)	0.14223 (2)	0.071476 (14)	0.02693 (7)	
Co1	0.5000	0.0000	0.0000	0.01023 (8)	
O1	0.36578 (13)	−0.11744 (14)	0.13111 (11)	0.0287 (3)	
H41	0.603 (2)	0.226 (3)	0.0534 (18)	0.037 (7)*	
H42	0.6254 (19)	0.163 (2)	−0.0114 (19)	0.030 (7)*	
O2	0.46952 (9)	0.04937 (13)	0.12000 (8)	0.0152 (3)	
O3	0.93287 (10)	−0.11547 (13)	0.39609 (9)	0.0169 (3)	
O4	0.61748 (10)	0.15516 (14)	0.03719 (10)	0.0142 (3)	
N1	0.64001 (11)	−0.12268 (15)	0.09116 (10)	0.0132 (3)	
N2	0.98258 (12)	0.00503 (16)	0.29417 (11)	0.0164 (3)	
C1	0.40749 (14)	−0.00794 (18)	0.15390 (13)	0.0150 (4)	
C2	0.38447 (14)	0.06833 (18)	0.22975 (13)	0.0158 (4)	
C3	0.28874 (15)	0.1439 (2)	0.20361 (13)	0.0187 (4)	
C4	0.27047 (16)	0.2238 (2)	0.27075 (15)	0.0247 (4)	
H4	0.2063	0.2751	0.2512	0.030*	
C5	0.34959 (17)	0.2251 (2)	0.36688 (15)	0.0275 (5)	
H5	0.3394	0.2790	0.4123	0.033*	
C6	0.44413 (16)	0.1466 (2)	0.39613 (15)	0.0264 (5)	
H6	0.4958	0.1456	0.4614	0.032*	
C7	0.46161 (15)	0.0693 (2)	0.32779 (14)	0.0217 (4)	
H7	0.5256	0.0176	0.3476	0.026*	
C8	0.72933 (13)	−0.07013 (18)	0.16504 (12)	0.0138 (4)	
H8	0.7295	0.0185	0.1752	0.017*	
C9	0.82096 (13)	−0.14176 (18)	0.22667 (12)	0.0129 (3)	
C10	0.82087 (13)	−0.27504 (19)	0.21376 (12)	0.0153 (4)	
H10	0.8800	−0.3260	0.2555	0.018*	
C11	0.72976 (14)	−0.32932 (19)	0.13661 (13)	0.0164 (4)	
H11	0.7275	−0.4177	0.1248	0.020*	
C12	0.64230 (13)	−0.25033 (18)	0.07735 (13)	0.0156 (4)	
H12	0.5822	−0.2878	0.0254	0.019*	
C13	0.91718 (13)	−0.08168 (18)	0.31162 (12)	0.0132 (4)	
C14	0.97403 (16)	0.0414 (2)	0.19635 (14)	0.0267 (5)	
H14A	0.9242	−0.0188	0.1483	0.032*	
H14B	1.0479	0.0345	0.1954	0.032*	
C15	0.92981 (17)	0.1775 (3)	0.16739 (17)	0.0392 (6)	
H15A	0.9321	0.1992	0.1058	0.059*	
H15B	0.9759	0.2371	0.2169	0.059*	
H15C	0.8535	0.1825	0.1611	0.059*	
C16	1.08067 (14)	0.0566 (2)	0.37816 (13)	0.0189 (4)	
H16A	1.0633	0.0604	0.4353	0.023*	
H16B	1.0961	0.1438	0.3634	0.023*	
C17	1.18452 (15)	−0.0267 (2)	0.40117 (15)	0.0256 (5)	
H17A	1.2461	0.0075	0.4575	0.038*	
H17B	1.2042	−0.0268	0.3460	0.038*	
H17C	1.1689	−0.1134	0.4146	0.038*	

### Atomic displacement parameters (Å^2^)

**Table:**

	*U*^11^	*U*^22^	*U*^33^	*U*^12^	*U*^13^	*U*^23^
Br1	0.02492 (10)	0.03337 (14)	0.02163 (10)	0.00625 (9)	0.00871 (7)	0.00300 (9)
Co1	0.01015 (13)	0.01029 (18)	0.00981 (15)	−0.00021 (13)	0.00367 (11)	−0.00054 (14)
O1	0.0486 (9)	0.0161 (8)	0.0359 (8)	−0.0111 (7)	0.0320 (7)	−0.0081 (7)
O2	0.0167 (5)	0.0160 (7)	0.0148 (6)	−0.0032 (5)	0.0084 (5)	−0.0023 (6)
O3	0.0195 (6)	0.0154 (7)	0.0119 (6)	−0.0016 (5)	0.0027 (5)	0.0017 (5)
O4	0.0166 (5)	0.0111 (7)	0.0152 (6)	0.0006 (5)	0.0069 (5)	0.0005 (6)
N1	0.0139 (6)	0.0131 (8)	0.0122 (7)	0.0000 (6)	0.0049 (5)	−0.0001 (6)
N2	0.0163 (6)	0.0176 (9)	0.0131 (7)	−0.0036 (6)	0.0040 (5)	0.0003 (7)
C1	0.0163 (7)	0.0141 (10)	0.0157 (8)	0.0017 (7)	0.0076 (6)	0.0007 (8)
C2	0.0208 (8)	0.0127 (10)	0.0178 (9)	−0.0038 (8)	0.0119 (7)	−0.0002 (8)
C3	0.0222 (8)	0.0189 (10)	0.0177 (9)	−0.0027 (8)	0.0109 (7)	−0.0003 (8)
C4	0.0302 (9)	0.0212 (11)	0.0310 (11)	0.0018 (9)	0.0209 (8)	−0.0018 (10)
C5	0.0403 (11)	0.0244 (12)	0.0278 (11)	−0.0071 (10)	0.0240 (9)	−0.0107 (10)
C6	0.0286 (9)	0.0330 (13)	0.0193 (9)	−0.0096 (9)	0.0116 (8)	−0.0070 (10)
C7	0.0191 (8)	0.0245 (12)	0.0218 (9)	−0.0035 (8)	0.0089 (7)	−0.0021 (9)
C8	0.0167 (7)	0.0112 (9)	0.0133 (8)	0.0005 (7)	0.0059 (6)	−0.0002 (7)
C9	0.0138 (7)	0.0132 (9)	0.0110 (8)	−0.0004 (7)	0.0044 (6)	0.0006 (8)
C10	0.0155 (7)	0.0143 (10)	0.0149 (8)	0.0044 (7)	0.0051 (6)	0.0042 (8)
C11	0.0199 (8)	0.0108 (9)	0.0169 (9)	−0.0002 (7)	0.0060 (7)	−0.0006 (8)
C12	0.0137 (7)	0.0160 (10)	0.0150 (8)	−0.0015 (7)	0.0037 (6)	−0.0023 (8)
C13	0.0133 (7)	0.0100 (9)	0.0135 (8)	0.0024 (7)	0.0026 (6)	−0.0004 (7)
C14	0.0255 (9)	0.0390 (14)	0.0146 (9)	−0.0125 (10)	0.0071 (7)	0.0024 (10)
C15	0.0280 (10)	0.0483 (16)	0.0315 (12)	−0.0094 (11)	0.0023 (9)	0.0222 (12)
C16	0.0179 (8)	0.0189 (11)	0.0161 (9)	−0.0066 (8)	0.0033 (7)	−0.0018 (8)
C17	0.0181 (8)	0.0306 (12)	0.0242 (10)	−0.0013 (9)	0.0049 (7)	0.0041 (9)

### Geometric parameters (Å, °)

**Table:**

Br1—C3	1.9053 (18)	C6—C7	1.388 (3)
Co1—O2	2.0559 (12)	C6—H6	0.9300
Co1—O2^i^	2.0559 (12)	C7—H7	0.9300
Co1—O4^i^	2.1272 (13)	C8—H8	0.9300
Co1—N1^i^	2.1783 (14)	C9—C8	1.384 (2)
O1—C1	1.242 (2)	C9—C10	1.393 (3)
O2—C1	1.264 (2)	C9—C13	1.503 (2)
O3—C13	1.244 (2)	C10—C11	1.388 (2)
O4—Co1	2.1272 (13)	C10—H10	0.9300
O4—H41	0.82 (3)	C11—H11	0.9300
O4—H42	0.78 (3)	C12—C11	1.385 (2)
N1—C12	1.340 (2)	C12—H12	0.9300
N1—C8	1.345 (2)	C14—C15	1.516 (3)
N1—Co1	2.1783 (14)	C14—H14A	0.9700
N2—C13	1.334 (2)	C14—H14B	0.9700
N2—C14	1.470 (2)	C15—H15A	0.9600
N2—C16	1.474 (2)	C15—H15B	0.9600
C2—C1	1.510 (2)	C15—H15C	0.9600
C2—C7	1.395 (2)	C16—C17	1.518 (3)
C3—C2	1.384 (3)	C16—H16A	0.9700
C3—C4	1.395 (3)	C16—H16B	0.9700
C4—C5	1.381 (3)	C17—H17A	0.9600
C4—H4	0.9300	C17—H17B	0.9600
C5—H5	0.9300	C17—H17C	0.9600
C6—C5	1.386 (3)		
			
O2—Co1—O2^i^	180.00 (5)	C6—C7—C2	120.88 (18)
O2—Co1—O4	87.71 (5)	C6—C7—H7	119.6
O2^i^—Co1—O4	92.29 (5)	N1—C8—C9	123.11 (17)
O2—Co1—O4^i^	92.29 (5)	N1—C8—H8	118.4
O2^i^—Co1—O4^i^	87.71 (5)	C9—C8—H8	118.4
O2—Co1—N1	90.60 (5)	C8—C9—C10	119.13 (15)
O2^i^—Co1—N1	89.40 (5)	C8—C9—C13	122.05 (16)
O2—Co1—N1^i^	89.40 (5)	C10—C9—C13	118.68 (15)
O2^i^—Co1—N1^i^	90.60 (5)	C9—C10—H10	121.1
O4^i^—Co1—O4	180.00 (7)	C11—C10—C9	117.90 (16)
O4—Co1—N1	87.18 (5)	C11—C10—H10	121.1
O4^i^—Co1—N1	92.82 (5)	C10—C11—H11	120.4
O4—Co1—N1^i^	92.82 (5)	C12—C11—C10	119.25 (18)
O4^i^—Co1—N1^i^	87.18 (5)	C12—C11—H11	120.4
N1^i^—Co1—N1	180.00 (13)	N1—C12—C11	123.24 (16)
C1—O2—Co1	128.18 (12)	N1—C12—H12	118.4
Co1—O4—H41	121.8 (17)	C11—C12—H12	118.4
Co1—O4—H42	100.9 (18)	O3—C13—N2	122.44 (15)
H42—O4—H41	109 (2)	O3—C13—C9	118.27 (15)
C8—N1—Co1	119.70 (12)	N2—C13—C9	119.29 (15)
C12—N1—Co1	122.99 (11)	N2—C14—C15	112.75 (18)
C12—N1—C8	117.32 (15)	N2—C14—H14A	109.0
C13—N2—C14	125.02 (15)	N2—C14—H14B	109.0
C13—N2—C16	118.37 (15)	C15—C14—H14A	109.0
C14—N2—C16	116.09 (14)	C15—C14—H14B	109.0
O1—C1—O2	126.67 (17)	H14A—C14—H14B	107.8
O1—C1—C2	118.94 (15)	C14—C15—H15A	109.5
O2—C1—C2	114.40 (16)	C14—C15—H15B	109.5
C3—C2—C1	121.13 (16)	C14—C15—H15C	109.5
C3—C2—C7	117.86 (17)	H15A—C15—H15B	109.5
C7—C2—C1	120.95 (16)	H15A—C15—H15C	109.5
C2—C3—Br1	119.59 (14)	H15B—C15—H15C	109.5
C2—C3—C4	122.13 (17)	N2—C16—C17	111.41 (16)
C4—C3—Br1	118.26 (14)	N2—C16—H16A	109.3
C3—C4—H4	120.6	N2—C16—H16B	109.3
C5—C4—C3	118.71 (19)	C17—C16—H16A	109.3
C5—C4—H4	120.6	C17—C16—H16B	109.3
C4—C5—C6	120.46 (18)	H16A—C16—H16B	108.0
C4—C5—H5	119.8	C16—C17—H17A	109.5
C6—C5—H5	119.8	C16—C17—H17B	109.5
C5—C6—C7	119.88 (18)	C16—C17—H17C	109.5
C5—C6—H6	120.1	H17A—C17—H17B	109.5
C7—C6—H6	120.1	H17A—C17—H17C	109.5
C2—C7—H7	119.6	H17B—C17—H17C	109.5
			
O4—Co1—O2—C1	−175.42 (14)	C3—C2—C1—O1	−86.0 (2)
O4^i^—Co1—O2—C1	4.58 (14)	C3—C2—C1—O2	94.2 (2)
N1—Co1—O2—C1	−88.27 (14)	C7—C2—C1—O1	96.9 (2)
N1^i^—Co1—O2—C1	91.73 (14)	C7—C2—C1—O2	−82.9 (2)
Co1—O2—C1—O1	12.3 (3)	C1—C2—C7—C6	175.44 (17)
Co1—O2—C1—C2	−167.89 (11)	C3—C2—C7—C6	−1.8 (3)
C8—N1—Co1—O2	−61.76 (13)	Br1—C3—C2—C1	4.6 (2)
C8—N1—Co1—O2^i^	118.24 (13)	Br1—C3—C4—C5	179.62 (15)
C8—N1—Co1—O4	25.92 (13)	Br1—C3—C2—C7	−178.19 (14)
C8—N1—Co1—O4^i^	−154.08 (13)	C2—C3—C4—C5	−1.4 (3)
C12—N1—Co1—O2	118.37 (14)	C4—C3—C2—C1	−174.33 (17)
C12—N1—Co1—O2^i^	−61.63 (14)	C4—C3—C2—C7	2.9 (3)
C12—N1—Co1—O4	−153.95 (14)	C3—C4—C5—C6	−1.2 (3)
C12—N1—Co1—O4^i^	26.05 (14)	C7—C6—C5—C4	2.2 (3)
Co1—N1—C8—C9	179.40 (13)	C5—C6—C7—C2	−0.7 (3)
Co1—N1—C12—C11	−178.31 (13)	C10—C9—C8—N1	−1.4 (3)
C8—N1—C12—C11	1.8 (3)	C13—C9—C8—N1	−177.05 (15)
C12—N1—C8—C9	−0.7 (2)	C8—C9—C10—C11	2.4 (3)
C14—N2—C13—O3	174.95 (18)	C13—C9—C10—C11	178.18 (15)
C14—N2—C13—C9	−4.8 (3)	C8—C9—C13—O3	114.69 (19)
C16—N2—C13—O3	3.6 (3)	C8—C9—C13—N2	−65.5 (2)
C16—N2—C13—C9	−176.22 (15)	C10—C9—C13—O3	−61.0 (2)
C13—N2—C14—C15	110.3 (2)	C10—C9—C13—N2	118.79 (19)
C13—N2—C16—C17	88.6 (2)	C9—C10—C11—C12	−1.4 (3)
C16—N2—C14—C15	−78.2 (2)	N1—C12—C11—C10	−0.8 (3)
C14—N2—C16—C17	−83.5 (2)		

Symmetry codes: (i) −*x*+1, −*y*, −*z*.

### Hydrogen-bond geometry (Å, °)

**Table:**

*D*—H···*A*	*D*—H	H···*A*	*D*···*A*	*D*—H···*A*
O4—H41···O3^ii^	0.82 (3)	1.94 (3)	2.757 (2)	174 (3)
O4—H42···O1^i^	0.78 (3)	1.90 (3)	2.644 (2)	159 (2)
C4—H4···O1^iii^	0.93	2.56	3.184 (3)	125
C10—H10···O2^iv^	0.93	2.44	3.368 (2)	174
C12—H12···O3^v^	0.93	2.33	3.259 (2)	179
C16—H16A···O3^vi^	0.97	2.57	3.506 (2)	163
C16—H16B···O1^ii^	0.97	2.52	3.460 (3)	164

Symmetry codes: (ii) −*x*+3/2, *y*+1/2, −*z*+1/2; (i) −*x*+1, −*y*, −*z*; (iii) −*x*+1/2, *y*+1/2, −*z*+1/2; (iv) −*x*+3/2, *y*−1/2, −*z*+1/2; (v) *x*−1/2, −*y*−1/2, *z*−1/2; (vi) −*x*+2, −*y*, −*z*+1.
